# 4-(5-Oxo-3-phenyl-4,5-dihydro-1*H*-pyrazol-1-yl)benzene­sulfonamide

**DOI:** 10.1107/S1600536811032879

**Published:** 2011-08-27

**Authors:** Abdulrahman O. Al-Youbi, Abdullah M. Asiri, Hassan M. Faidallah, Khalid A. Alamry, Seik Weng Ng

**Affiliations:** a Chemistry Department, Faculty of Science, King Abdulaziz University, PO Box 80203 Jeddah, Saudi Arabia; b Center of Excellence for Advanced Materials Research, King Abdulaziz University, PO Box 80203 Jeddah, Saudi Arabia; c Department of Chemistry, University of Malaya, 50603 Kuala Lumpur, Malaysia

## Abstract

With respect to the aliphatic planar five-membered ring (r.m.s. deviation = 0.011 Å) of the title compound, C_15_H_13_N_3_O_2_S, the phenyl ring is aligned at 6.9 (1)° and the phenyl­ene ring at 2.4 (1)°, so that the three rings are nearly coplanar. The amino group has the N atom in a pyramidal geometry; the group is a hydrogen-bond donor to the sulfonyl O atom of one mol­ecule and to the ketonic O atom of another mol­ecule, resulting in the formation of a layer parallel to the *bc* plane.

## Related literature

For the synthesis, see: Casoni (1956 ▶); Itano (1955 ▶).
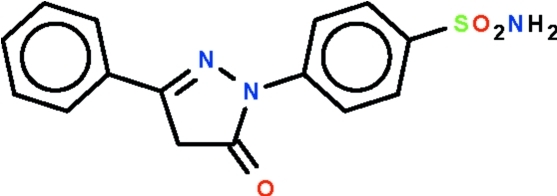


## Experimental

### 

#### Crystal data

C_15_H_13_N_3_O_3_S
*M* *_r_* = 315.34
Monoclinic, 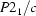

*a* = 13.6794 (4) Å
*b* = 13.4304 (4) Å
*c* = 7.3678 (2) Å
β = 91.055 (3)°
*V* = 1353.38 (7) Å^3^
*Z* = 4
Cu *K*α radiation
μ = 2.29 mm^−1^
*T* = 100 K
0.30 × 0.05 × 0.05 mm

#### Data collection

Agilent SuperNova Dual diffractometer with an Atlas detector
Absorption correction: multi-scan (*CrysAlis PRO*; Agilent, 2010 ▶) *T*_min_ = 0.546, *T*_max_ = 0.894
10404 measured reflections
2731 independent reflections
2444 reflections with *I* > 2σ(*I*)
*R*_int_ = 0.041

#### Refinement

*R*[*F* ^2^ > 2σ(*F* ^2^)] = 0.041
*wR*(*F* ^2^) = 0.121
*S* = 1.06
2731 reflections
207 parameters
2 restraints
H atoms treated by a mixture of independent and constrained refinement
Δρ_max_ = 0.73 e Å^−3^
Δρ_min_ = −0.51 e Å^−3^

### 

Data collection: *CrysAlis PRO* (Agilent, 2010 ▶); cell refinement: *CrysAlis PRO*; data reduction: *CrysAlis PRO*; program(s) used to solve structure: *SHELXS97* (Sheldrick, 2008 ▶); program(s) used to refine structure: *SHELXL97* (Sheldrick, 2008 ▶); molecular graphics: *X-SEED* (Barbour, 2001 ▶); software used to prepare material for publication: *publCIF* (Westrip, 2010 ▶).

## Supplementary Material

Crystal structure: contains datablock(s) global, I. DOI: 10.1107/S1600536811032879/bt5611sup1.cif

Structure factors: contains datablock(s) I. DOI: 10.1107/S1600536811032879/bt5611Isup2.hkl

Supplementary material file. DOI: 10.1107/S1600536811032879/bt5611Isup3.cml

Additional supplementary materials:  crystallographic information; 3D view; checkCIF report

## Figures and Tables

**Table 1:** Hydrogen-bond geometry (Å, °)

*D*—H⋯*A*	*D*—H	H⋯*A*	*D*⋯*A*	*D*—H⋯*A*
N3—H1⋯O3^i^	0.88 (1)	2.12 (1)	2.975 (2)	164 (2)
N3—H2⋯O2^ii^	0.87 (1)	2.12 (1)	2.978 (2)	168 (2)

Symmetry codes: (i) 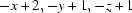; (ii) 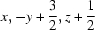.

**Fig. 1.**
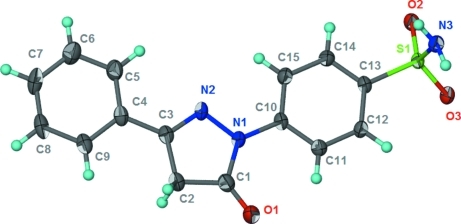
Anisotropic displacement ellipsoid plot (Barbour, 2001) of C15H13N3O2S at the 70% probability level; hydrogen atoms are drawn as spheres of arbitrary radius.

## supplementary crystallographic information

### Comment

We are examining the medicinal properties of phenylpyrazolones of which the 4-benzenesulfamide derivative (Scheme I) is expected to show enhanced activity. The synthesis of the compound was reported a long time ago (Casoni, 1956; Itano, 1955). With respect to the aliphatic planar five-membered ring, the phenyl ring is aligned at 6.9 (1)° and the phenylene ring at 2.4 (1)°. The amino group is hydrogen bond donor to the sulfonyl O atom of one molecule and to the ketonic O atom of another molecule to result in the formation of a layer parallel to  the bc plane.

### Experimental

Ethyl benzoylacetate (10 mmol) and 4-hydrazinobenzenesulfonamide hydrochloride (10 mmol) were heated in ethanol (50 ml) for 4 h; water was added to precipitate the product, which was collected and recrystallized from ethanol as brownish-orange crystals; m.p. 510–511 K.

### Refinement

Carbon bound H-atoms were placed in calculated positions [C–H 0.95 to 0.98 Å, *U*_iso_(H) 1.2–1.5*U*_eq_(C)] and were included in the refinement in the riding model approximation.

The amino H-atoms were located in a difference Fouier map and were refined with a distance restraint of N–H 0.88±0.01 Å; their isotropic displacement parameter were freely refined.

### Crystal data

**Table:**

C_15_H_13_N_3_O_3_S	*F*(000) = 656
*M**_r_* = 315.34	*D*_x_ = 1.548 Mg m^−^^3^
Monoclinic, *P*2_1_/*c*	Cu *K*α radiation, λ = 1.54184 Å
Hall symbol: -P 2ybc	Cell parameters from 4563 reflections
*a* = 13.6794 (4) Å	θ = 3.2–74.4°
*b* = 13.4304 (4) Å	µ = 2.29 mm^−^^1^
*c* = 7.3678 (2) Å	*T* = 100 K
β = 91.055 (3)°	Prism, brown orange
*V* = 1353.38 (7) Å^3^	0.30 × 0.05 × 0.05 mm
*Z* = 4	

### Data collection

**Table:**

Agilent SuperNova Dual diffractometer with an Atlas detector	2731 independent reflections
Radiation source: SuperNova (Cu) X-ray Source	2444 reflections with *I* > 2σ(*I*)
Mirror	*R*_int_ = 0.041
Detector resolution: 10.4041 pixels mm^-1^	θ_max_ = 74.5°, θ_min_ = 3.2°
ω scan	*h* = −17→16
Absorption correction: multi-scan (*CrysAlis PRO*; Agilent, 2010)	*k* = −16→16
*T*_min_ = 0.546, *T*_max_ = 0.894	*l* = −5→9
10404 measured reflections	

### Refinement

**Table:**

Refinement on *F*^2^	Primary atom site location: structure-invariant direct methods
Least-squares matrix: full	Secondary atom site location: difference Fourier map
*R*[*F*^2^ > 2σ(*F*^2^)] = 0.041	Hydrogen site location: inferred from neighbouring sites
*wR*(*F*^2^) = 0.121	H atoms treated by a mixture of independent and constrained refinement
*S* = 1.06	*w* = 1/[σ^2^(*F*_o_^2^) + (0.0759*P*)^2^ + 0.5321*P*] where *P* = (*F*_o_^2^ + 2*F*_c_^2^)/3
2731 reflections	(Δ/σ)_max_ = 0.001
207 parameters	Δρ_max_ = 0.73 e Å^−^^3^
2 restraints	Δρ_min_ = −0.51 e Å^−^^3^

### Special details

**Table:**

Geometry. All e.s.d.'s (except the e.s.d. in the dihedral angle between two l.s. planes) are estimated using the full covariance matrix. The cell e.s.d.'s are taken into account individually in the estimation of e.s.d.'s in distances, angles and torsion angles; correlations between e.s.d.'s in cell parameters are only used when they are defined by crystal symmetry. An approximate (isotropic) treatment of cell e.s.d.'s is used for estimating e.s.d.'s involving l.s. planes.

### Fractional atomic coordinates and isotropic or equivalent isotropic displacement parameters (Å^2^)

**Table:**

	*x*	*y*	*z*	*U*_iso_*/*U*_eq_	
S1	0.91654 (3)	0.64477 (3)	0.49527 (6)	0.01653 (16)	
O1	0.52785 (10)	0.32661 (11)	0.6467 (2)	0.0319 (4)	
O2	0.90632 (9)	0.74972 (10)	0.46298 (18)	0.0220 (3)	
O3	0.95114 (9)	0.58212 (10)	0.35168 (17)	0.0209 (3)	
N2	0.46514 (11)	0.56244 (12)	0.8066 (2)	0.0203 (3)	
N3	0.99249 (12)	0.63163 (12)	0.6642 (2)	0.0197 (3)	
H1	1.0122 (17)	0.5704 (9)	0.682 (3)	0.028 (6)*	
H2	0.9740 (16)	0.6620 (16)	0.763 (2)	0.023 (6)*	
N1	0.52929 (11)	0.49471 (11)	0.7246 (2)	0.0197 (3)	
C1	0.49011 (14)	0.39912 (14)	0.7147 (3)	0.0227 (4)	
C2	0.39264 (14)	0.40640 (14)	0.8033 (3)	0.0224 (4)	
H2A	0.3390	0.3864	0.7189	0.027*	
H2B	0.3903	0.3645	0.9138	0.027*	
C3	0.38673 (13)	0.51403 (14)	0.8491 (2)	0.0198 (4)	
C4	0.30272 (13)	0.56293 (14)	0.9324 (2)	0.0205 (4)	
C5	0.30792 (15)	0.66235 (15)	0.9877 (3)	0.0261 (4)	
H5	0.3663	0.6993	0.9713	0.031*	
C6	0.22764 (16)	0.70694 (16)	1.0664 (3)	0.0312 (5)	
H6	0.2311	0.7745	1.1040	0.037*	
C7	0.14192 (15)	0.65297 (16)	1.0905 (3)	0.0294 (5)	
H7	0.0870	0.6837	1.1441	0.035*	
C8	0.13695 (14)	0.55477 (17)	1.0363 (3)	0.0281 (5)	
H8	0.0784	0.5181	1.0528	0.034*	
C9	0.21695 (13)	0.50911 (15)	0.9577 (3)	0.0235 (4)	
H9	0.2132	0.4413	0.9213	0.028*	
C10	0.62051 (12)	0.52987 (13)	0.6675 (2)	0.0178 (4)	
C11	0.68801 (13)	0.46447 (14)	0.5912 (2)	0.0197 (4)	
H11	0.6720	0.3961	0.5759	0.024*	
C12	0.77831 (13)	0.49990 (13)	0.5381 (2)	0.0184 (4)	
H12	0.8246	0.4557	0.4875	0.022*	
C13	0.80101 (13)	0.60030 (13)	0.5590 (2)	0.0175 (4)	
C14	0.73339 (14)	0.66554 (14)	0.6338 (3)	0.0201 (4)	
H14	0.7495	0.7339	0.6481	0.024*	
C15	0.64299 (14)	0.63125 (14)	0.6873 (3)	0.0200 (4)	
H15	0.5967	0.6759	0.7369	0.024*	

### Atomic displacement parameters (Å^2^)

**Table:**

	*U*^11^	*U*^22^	*U*^33^	*U*^12^	*U*^13^	*U*^23^
S1	0.0140 (2)	0.0175 (3)	0.0182 (3)	−0.00041 (14)	0.00381 (17)	0.00075 (14)
O1	0.0230 (7)	0.0225 (7)	0.0506 (9)	−0.0015 (6)	0.0103 (7)	−0.0049 (6)
O2	0.0202 (6)	0.0184 (7)	0.0276 (7)	−0.0010 (5)	0.0052 (5)	0.0040 (5)
O3	0.0194 (6)	0.0241 (7)	0.0194 (6)	−0.0005 (5)	0.0061 (5)	−0.0021 (5)
N2	0.0148 (7)	0.0241 (8)	0.0221 (8)	0.0014 (6)	0.0047 (6)	−0.0003 (6)
N3	0.0180 (8)	0.0205 (8)	0.0208 (8)	0.0015 (6)	0.0012 (6)	−0.0009 (6)
N1	0.0161 (7)	0.0206 (8)	0.0226 (8)	−0.0004 (6)	0.0050 (6)	−0.0023 (6)
C1	0.0194 (9)	0.0227 (10)	0.0259 (9)	−0.0017 (7)	0.0013 (7)	0.0021 (7)
C2	0.0179 (9)	0.0251 (10)	0.0242 (9)	−0.0018 (7)	0.0024 (7)	0.0015 (7)
C3	0.0168 (9)	0.0248 (9)	0.0178 (8)	−0.0014 (7)	0.0008 (7)	0.0022 (7)
C4	0.0162 (9)	0.0274 (9)	0.0179 (8)	0.0009 (7)	0.0022 (7)	0.0049 (7)
C5	0.0236 (10)	0.0259 (10)	0.0290 (10)	0.0004 (8)	0.0083 (8)	0.0056 (8)
C6	0.0326 (11)	0.0261 (10)	0.0353 (11)	0.0068 (8)	0.0120 (9)	0.0078 (8)
C7	0.0220 (10)	0.0400 (12)	0.0266 (10)	0.0123 (8)	0.0080 (8)	0.0114 (8)
C8	0.0153 (9)	0.0450 (12)	0.0241 (10)	−0.0017 (8)	0.0019 (7)	0.0068 (8)
C9	0.0180 (9)	0.0329 (10)	0.0195 (9)	−0.0034 (8)	0.0007 (7)	0.0007 (7)
C10	0.0146 (8)	0.0220 (9)	0.0170 (8)	−0.0006 (7)	0.0012 (6)	0.0019 (7)
C11	0.0190 (9)	0.0203 (9)	0.0200 (8)	−0.0009 (7)	0.0019 (7)	−0.0004 (7)
C12	0.0161 (8)	0.0193 (8)	0.0199 (9)	0.0024 (7)	0.0029 (7)	0.0004 (6)
C13	0.0141 (8)	0.0208 (9)	0.0178 (8)	−0.0012 (7)	0.0015 (6)	0.0019 (6)
C14	0.0195 (9)	0.0171 (8)	0.0240 (9)	−0.0002 (7)	0.0033 (7)	0.0000 (7)
C15	0.0177 (9)	0.0211 (9)	0.0213 (9)	0.0017 (7)	0.0029 (7)	−0.0007 (7)

### Geometric parameters (Å, °)

**Table:**

S1—O2	1.4358 (13)	C5—H5	0.9500
S1—O3	1.4386 (13)	C6—C7	1.393 (3)
S1—N3	1.6160 (17)	C6—H6	0.9500
S1—C13	1.7611 (18)	C7—C8	1.379 (3)
O1—C1	1.215 (2)	C7—H7	0.9500
N2—C3	1.298 (2)	C8—C9	1.390 (3)
N2—N1	1.408 (2)	C8—H8	0.9500
N3—H1	0.875 (10)	C9—H9	0.9500
N3—H2	0.873 (10)	C10—C11	1.400 (2)
N1—C1	1.393 (2)	C10—C15	1.403 (3)
N1—C10	1.406 (2)	C11—C12	1.387 (3)
C1—C2	1.499 (3)	C11—H11	0.9500
C2—C3	1.487 (3)	C12—C13	1.392 (2)
C2—H2A	0.9900	C12—H12	0.9500
C2—H2B	0.9900	C13—C14	1.395 (2)
C3—C4	1.468 (2)	C14—C15	1.384 (3)
C4—C9	1.393 (3)	C14—H14	0.9500
C4—C5	1.397 (3)	C15—H15	0.9500
C5—C6	1.387 (3)		
			
O2—S1—O3	119.00 (8)	C5—C6—C7	120.2 (2)
O2—S1—N3	107.10 (8)	C5—C6—H6	119.9
O3—S1—N3	106.67 (8)	C7—C6—H6	119.9
O2—S1—C13	106.97 (8)	C8—C7—C6	119.86 (19)
O3—S1—C13	107.85 (8)	C8—C7—H7	120.1
N3—S1—C13	108.97 (8)	C6—C7—H7	120.1
C3—N2—N1	107.72 (15)	C7—C8—C9	120.49 (19)
S1—N3—H1	114.1 (16)	C7—C8—H8	119.8
S1—N3—H2	113.3 (16)	C9—C8—H8	119.8
H1—N3—H2	114 (2)	C8—C9—C4	119.88 (19)
C1—N1—C10	129.54 (15)	C8—C9—H9	120.1
C1—N1—N2	112.08 (14)	C4—C9—H9	120.1
C10—N1—N2	118.38 (14)	C11—C10—N1	120.35 (16)
O1—C1—N1	126.49 (17)	C11—C10—C15	120.39 (16)
O1—C1—C2	128.35 (18)	N1—C10—C15	119.26 (16)
N1—C1—C2	105.17 (16)	C12—C11—C10	119.71 (17)
C3—C2—C1	102.40 (15)	C12—C11—H11	120.1
C3—C2—H2A	111.3	C10—C11—H11	120.1
C1—C2—H2A	111.3	C11—C12—C13	119.97 (16)
C3—C2—H2B	111.3	C11—C12—H12	120.0
C1—C2—H2B	111.3	C13—C12—H12	120.0
H2A—C2—H2B	109.2	C12—C13—C14	120.26 (16)
N2—C3—C4	122.26 (17)	C12—C13—S1	119.91 (13)
N2—C3—C2	112.57 (16)	C14—C13—S1	119.81 (14)
C4—C3—C2	125.17 (16)	C15—C14—C13	120.41 (17)
C9—C4—C5	119.68 (17)	C15—C14—H14	119.8
C9—C4—C3	119.44 (17)	C13—C14—H14	119.8
C5—C4—C3	120.88 (17)	C14—C15—C10	119.25 (17)
C6—C5—C4	119.85 (19)	C14—C15—H15	120.4
C6—C5—H5	120.1	C10—C15—H15	120.4
C4—C5—H5	120.1		
			
C3—N2—N1—C1	−0.1 (2)	C5—C4—C9—C8	0.5 (3)
C3—N2—N1—C10	−179.52 (16)	C3—C4—C9—C8	−179.92 (17)
C10—N1—C1—O1	−2.7 (3)	C1—N1—C10—C11	−1.6 (3)
N2—N1—C1—O1	177.9 (2)	N2—N1—C10—C11	177.72 (15)
C10—N1—C1—C2	177.83 (17)	C1—N1—C10—C15	178.21 (18)
N2—N1—C1—C2	−1.6 (2)	N2—N1—C10—C15	−2.4 (2)
O1—C1—C2—C3	−177.1 (2)	N1—C10—C11—C12	−178.97 (16)
N1—C1—C2—C3	2.34 (19)	C15—C10—C11—C12	1.2 (3)
N1—N2—C3—C4	−178.03 (16)	C10—C11—C12—C13	−0.7 (3)
N1—N2—C3—C2	1.7 (2)	C11—C12—C13—C14	0.2 (3)
C1—C2—C3—N2	−2.6 (2)	C11—C12—C13—S1	178.85 (14)
C1—C2—C3—C4	177.17 (17)	O2—S1—C13—C12	157.60 (15)
N2—C3—C4—C9	173.44 (17)	O3—S1—C13—C12	28.50 (17)
C2—C3—C4—C9	−6.3 (3)	N3—S1—C13—C12	−86.93 (16)
N2—C3—C4—C5	−7.0 (3)	O2—S1—C13—C14	−23.78 (17)
C2—C3—C4—C5	173.27 (18)	O3—S1—C13—C14	−152.88 (15)
C9—C4—C5—C6	−0.3 (3)	N3—S1—C13—C14	91.69 (16)
C3—C4—C5—C6	−179.88 (18)	C12—C13—C14—C15	−0.3 (3)
C4—C5—C6—C7	0.0 (3)	S1—C13—C14—C15	−178.89 (14)
C5—C6—C7—C8	0.2 (3)	C13—C14—C15—C10	0.8 (3)
C6—C7—C8—C9	0.0 (3)	C11—C10—C15—C14	−1.2 (3)
C7—C8—C9—C4	−0.4 (3)	N1—C10—C15—C14	178.94 (16)

### Hydrogen-bond geometry (Å, °)

**Table:**

*D*—H···*A*	*D*—H	H···*A*	*D*···*A*	*D*—H···*A*
N3—H1···O3^i^	0.88 (1)	2.12 (1)	2.975 (2)	164 (2)
N3—H2···O2^ii^	0.87 (1)	2.12 (1)	2.978 (2)	168 (2)

Symmetry codes: (i) −*x*+2, −*y*+1, −*z*+1; (ii) *x*, −*y*+3/2, *z*+1/2.
